# Development of Photocatalytic Coatings by Thermal Hydrolysis of TiCl_4_ on Ceramic Roofing Tiles Made from Ferroalumina and Evaluation of De-Pollution Properties

**DOI:** 10.3390/ma13030620

**Published:** 2020-01-30

**Authors:** Angeliki Christogerou, Dimitra Koumpouri, George N. Angelopoulos

**Affiliations:** 1 Department of Chemical Engineering, University of Patras, Caratheodori 1, 26504 Patras, Greece; angel@upatras.gr; 2 INVALOR: Research Infrastructure for Waste Valorization and Sustainable Management, Caratheodory 1, University Campus, 26504 Patras, Greece; 3 Institute of Nanoscience & Nanotechnology, National Center for Scientific Research Demokritos, Agia Paraskevi, 15342 Athens, Greece; d.koumpouri@inn.demokritos.gr

**Keywords:** clay roofing tiles, industrial by-product, ferroalumina, chemical vapor deposition, hydrolysis of TiCl_4_, TiO_2_ coatings, dye degradation, circular economy, antipollution

## Abstract

The development of new, environmental friendly building materials with photocatalytic properties remain still on the top of the investigations both for academy and industry. The main drive is the increasing air pollution and the greenhouse gas emissions that have negative effect on public health and buildings. Ceramic roofing tiles functionalized with TiO_2_ can contribute on tackling these severe environmental problems by improving their properties. In this study, heavy clay ceramics manufactured from clay-body mixture and a Bayer process bauxite residue (ferroalumina) are used as substrates for the deposition of TiO_2_ coatings in order to produce self-cleaning ceramic surfaces. The process is based on the thermal hydrolysis of TiCl_4_ which takes place in a CVD reactor under atmospheric conditions. All coated samples were annealed at 600 °C and characterized in means of XRD, SEM/EDS and degradation ability of an organic pollutant. The formation of titania mixed phases (rutile and perovskite) shows positive results regarding the photocatalytic activity of the samples. The ones containing ferroalumina decomposed 100% the indigo carmine solution after 4 h, in comparison with the reference one which presented lower efficiency. Finally, this paper addresses technical feasible solutions for the production of photocatalytic active ceramics within the concept of circular economy and environmental sustainability.

## 1. Introduction

The production of “smart” building materials and coatings designed on the basis of TiO_2_ has gained special attention in the last decade. In particular, the development of new technologies with regards to titania is targeted on self-cleaning surfaces and environmental applications. In this aspect, traditional ceramics such as roofing tiles are considered as good candidates for novel applications by enhancing their conventional properties into new ones with photocatalytic features [1,2]. This can be achieved through the development of functional coatings that consist of metal oxides such as titanium dioxide (TiO_2_). The later oxide has been proven to be an ideal photocatalyst in many fields of everyday applications like cosmetics, food, paints, ceramic glazes, textiles, and even in medicine [3,4] due to its excellent properties. 

One of these properties that attract great interest is the ability of TiO_2_ to participate in red-ox reactions which are activated under UV irradiation [5,6,7] leading to substantial reduction of airborne pollutants such as volatile organic compounds and nitrogen oxides (NO_x_ gases). The fundamental mechanism that takes place in heterogeneous photocatalysis with titania after irradiated by light (*hv* ≥ E_g_ = 3.2 eV) is the production of a reactive electron (e^−^)/hole (h^+^) pair (TiO_2_ + *hv* → TiO_2_ + h^+^ + e^−^). The photogenerated electrons (e^−^) can react with oxygen molecules (O_2_) from the atmosphere to form superoxide radicals (∙O_2_^−^). Accordingly, the positively charged surface of TiO_2_ (h^+^) can not only directly oxidize organic compounds due to its strong oxidizing ability, but also react with water molecules (moisture from the air) to form hydroxyl radical (∙OH) [8]. These active species can then decompose adsorbed organic pollutants to CO_2_ and water [2,9].

Another positive aspect of the heterogeneous photocatalysis with TiO_2_ are the conditions under which the reaction can take place, i.e., low level of UV radiation, room temperature and atmospheric pressure. Moreover, no chemical additives are necessary and the possible intermediates of the reactions are not dangerous or at least less dangerous than the original pollutant, even every recalcitrant and persistent pollutants can be degraded [3,10]. Thus, taking into account that UV radiation is present in daylight, and even on cloudy days, makes the TiO_2_ coatings very attractive to outdoor building materials. In addition, the aesthetic result of coated surfaces remains unaffected in case of nano-crystalline thin films that are transparent and colorless [11]. However, the photocatalytic activity of TiO_2_ is affected by different factors such as production techniques and conditions of preparation, surface properties and TiO_2_ phases (crystallinity, crystallite size, and symmetry). 

Regarding the production of titania nanoparticles and the related deposition techniques numerous scientific reports are found in the open literature. For example, it is stated in [12] that the nanocrystallinity of titania is strictly connected with the enhancing of the photocatalytic activity, due to the fact that it favors the diffusion on the surface of free electron and holes and limits their recombination through charge carrier trapping. Additionally, it has been observed that anatase (A) or mixture phases (anatase/rutile) present higher photoactivity than the pure crystalline phases of TiO_2_ like rutile (R) and brookite [1,13,14]. Zeatoun et al. [15] produced submicron TiO_2_ smoke by hydrolysis of TiCl_4_ at reactor temperatures between 210 °C and 817 °C. Higher reactor temperatures (405–817 °C) favor the formation of anatase, while amorphous powders resulted on lower reactor temperatures. Watanabe et al. [16] report that the activity of the photocatalytic fabricated tiles increases with the firing temperature up to 700 °C and presents the opposite effect when sintered at higher temperatures due to the transformation of anatase to rutile. In addition, TiO_2_ films loaded with metal additives (CuO) remarkably enhance their photocatalytic activity after firing at 900 °C. Sun et al. [17] prepared TiO_2_ films on glass by CVD at atmospheric pressure using TiCl_4_ as a precursor. They found that the ideal deposition is obtained when the spacing between nozzle and glass is about 3 mm. The deposition rate was found to increase with the substrate temperature. Anatase was detected as major crystalline phase and the degeneration efficiency of methyl orange increases with increase of deposition temperature. Song et al. [18] studied the deposition TiO_2_ on muscovite flakes doped with different metal ions using chemical liquid deposition method. Some metal ions (Mn^2+^, Sn^4+^, Fe^3+^ and Zn^2+^) promote the A-R transformation whilst others (Al^3+^, Cu^2+^, Co^2+^) inhibit it. Calcination temperature influences as well the A-R transformation which starts at 800 °C and is completed at 1000 °C. Ding et al. [19] studied the synthesis of anatase TiO_2_ supported on three different porous solids by CVD and evaluated their photocatalytic activity. Silica gel was found to be the most efficient support in terms of both anatase formation and photocatalytic reaction. 

In recent years, studies on new types of semiconductor catalysts have gained also remarkable interest as for perovskite compounds. Perovskite (CaTiO_3_) with the general formula ABO_3_ is frequently encountered structure in inorganic chemistry and can accommodate most of the metallic ions in the periodic table with a significant number of different anions. According to those studies perovskite can be used as a catalyst [6,20,21]. 

The evaluation of photocatalytic coatings involves a number of analyses; the most frequent used are SEM (microstructure, grain size and shape of particles), XRD (crystalline phases) and degradation of dyes giving valuable information about the materials performance. However, it is reported [22] that the evaluation of self-cleaning properties and photocatalytic degradation of pigments, concretes or non-transparent materials are limited in means of scientific literature and standard methods, compared to coated glasses which are commercially available. Nevertheless, the self-cleaning ability of opaque and rough surfaces is measured by reflectance spectroscopy as defined by the European standard EN 16845-1:2017 [23]. 

Despite the large number of studies available in the open literature concerning the production of TiO_2_ coatings on different supports, only few are focused on the production of photocatalytic heavy clay materials. Tobaldi et al. [3] investigated the addition of TiO_2_ powders in a clay body mix suitable for the production of heavy clay products and examined the factors which influence their photocatalytic activity. Moreover, Ducman et al. [2] studied the formation of photocatalytic coatings on ceramics building products (tiles, glass and roofing tiles) by spraying of a TiO_2_ isopropanol suspension. Another scientific work related to TiO_2_ functional tiles was performed by Vaiano et al. [24] who examined the influence of the substrates porosity on the photocatalytic activity of coated ceramic tiles. However, no work was found, up to now, in the literature regarding the application of Chemical Vapour Deposition (CVD) for the production of coated heavy clay ceramics.

In the current study, the development of photocatalytic active TiO_2_ spots on ceramic roofing tiles were studied using CVD as a coating method. For this purpose, feasibility tests were conducted, in laboratory scale, by depositing TiCl_4_ as a precursor solution on ceramic samples. The photocatalytic properties of the coated and annealed surfaces were successfully examined by XRD, SEM/EDS and decolorization ability of an indigo carmine solution. Finally, the technological outcomes of this work may be used as a basis for large-scale investigations, with the future perspective of an industrial scale application. 

## 2. Materials and Methods 

### 2.1. Ceramic Substrates 

**Figure 1:**
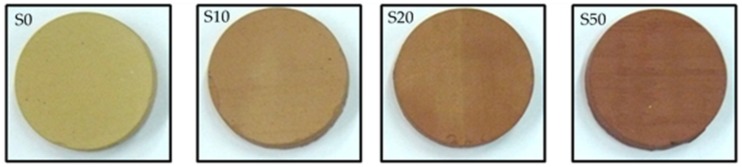
Fired (1000 °C) ceramic substrates with different amounts of ferroalumina. S0 (0 wt% FA), S10 (10 wt% FA), S20 (20 wt% FA).

The ceramic substrates used for the deposition of TiO_2_ are shown in Figure 1. They consist mainly of a calcareous rich Greek clay mixture (W) and ferroalumina (FA) in different percentages. Ferroalumina is an industrial by-product that derives from the dewatering of red mud, a bauxite residue of the Bayer’s process [25]. For the current investigation cylindrical samples (d = 5 cm, h = 1 cm) were cut off by a diamond hollow drill from rectangular ceramic samples fired at 1000 °C and named according to the included percentage of FA such as S0 (0 wt%), S10 (10 wt%), S20 (20 wt%), and S50 (50 wt%). The experimental procedure for their manufacture is described elsewhere [26]. 

### 2.2. Characterization Techniques

The chemical composition of the raw materials was determined by Atomic Absorption Spectrometer (AAnalyst 200, Perkin Elmer, Shelton, CT, USA). The mineralogical characterization of the coated surfaces was carried out by X-ray diffraction analysis (Siemens Diffraktometer D5000, Karlsruhe, Germany) in the 10–70°, 2*θ* range with a step size of 0.01° and a speed of 1 °/min.For the XRD measurements, Cu Kα radiation at 40 kV and 30 mA was used. The crystalline phases identified using the DIFFRACplus EVA12® software (Bruker-AXS, Billerica, MA, USA) based on the ICDD powder diffraction file of PDF-2 2006. The mineralogical phases were quantified with the Rietveld-based routine using the TOPAS® software (Diffrac*^Plus^* Professional, Version 4.2, Bruker-AXS, Karlsruhe, Germany). The morphology and size of the titania particles were analyzed by SEM/EDS (JSM-6300, Jeol, Tokyo, Japan) equipped with an X-ray energy dispersive spectrometer on gold coated surfaces.

### 2.3. CVD Apparatus and Procedures 

**Figure 2:**
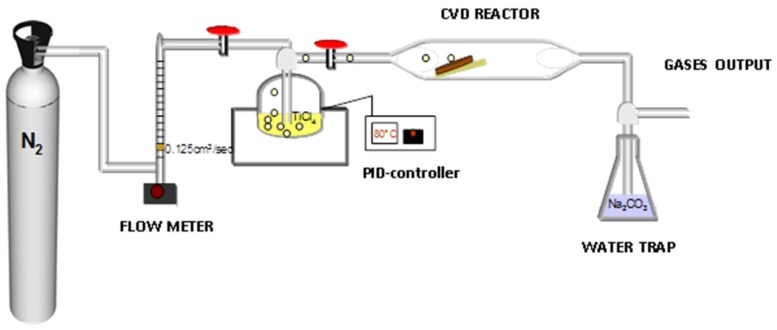
Schematic representation of the experimental CVD setup.

For the CVD process, a small experimental set-up was constructed and is depicted in Figure 2. TiCl_4_ (99.9% purity, Acros Organics^TM^, New Jersey, NJ, USA) was used as a precursor solution to form TiO_2_ spots on the ceramics’ surface. The liquid TiCl_4_ was placed in a spherical glass vessel within a nitrogen filled glove box in order to prevent vigorous reaction with moisture in the air. During the experiment, the glass vessel was heated by an electric heating mantel and the external temperature of the flask was measured using a K type thermocouple connected to a PID temperature controller. Nitrogen (99.9%) was used as a carrier gas to transport the vaporized precursor to the reactor chamber (cylindrical quartz tube) with a flow rate of 0.125 cm^3^/s. This rate results in a sufficient inflow of TiCl_4_ in order to achieve the formation of small size (sub-micron scale) TiO_2_ particles on the surface of the ceramics providing high reactive surface as well as the desirable adhesion with the substrate. Additionally, the used flow rate leads to the creation of an invisible TiO_2_ coating which does not affect the natural appearance of the ceramics. It was observed from our preliminary tests that higher inflow rates of the precursor led to the formation of a liquid gel which upon drying turned to a greenish powder attributed to chlorine contamination. Consequently, the formed coating was fragile and presented poor adhesion with the substrate. Similar behaviour is reported also elsewhere [19]. Wet scrubbing was employed at the outlet of the reactor using a caustic trap (Na_2_CO_3_ solution) for the removal of residual TiCl_4_ and other vapor by-products. 

The ceramic samples were sprayed with a solution containing Cu^2+^ ions and then placed in a suitable support at an angle of 20°, which was 15 cm approximately far from the nozzle. Metal ions are known to enhance both the crystallinity of TiO_2_ and the photoactivity of the final coating [16]. The temperature of the liquid TiCl_4_ was maintained at 60–80 °C during the whole process. The experimental procedure was carried out under atmospheric pressure and the deposition lasted for one hour. After the CVD process, the coated samples were annealed up to 600 °C in a laboratory muffle furnace for 1 hour and a heating rate of 20 °C/min. 

### 2.4. Photocatalytic Degradation Experiments 

The photocatalytic activity of the coated samples was evaluated by the degradation ability of an Indigo Carmine (IC) solution under UV irradiation. The organic dye was purchased from ACROS Organics^TM^ (New Jersey, NJ, USA). A stock solution (100 ppm) was prepared by dissolving and diluting 50 mg of Indigo Carmine to 500 mL in deionized water (Millipore, Direct Q-3, Molsheim, France). For the experiments, a cylindrical glass vessel was filled with 500 mL of the test solution (1 ppm). To ensure continuous flow of the liquid and removal of the surface reactants magnetically stirring was employed during the whole process. The coated substrate was placed in a suitable base; 10 mm under the water’s surface in order to be near the light source. UV irradiation was provided by two parallel blacklight blue lamps (6 W, Sen-lite, F6T5/BLB) with a wavelength of λ = 365 nm, which were placed horizontally above the sample. The light intensity reaching the samples surface was measured at 10 W/m^2^. The tests were carried out under ambient conditions at room temperature. 

Prior to all photocatalytic experiments, the IC solution was magnetically stirred for 30 min in dark in order to achieve adsorption equilibrium [6]. During irradiation, sampling from the reacting system took place every one hour. The obtained liquid was filtered through a 0.45 μm Porafil filter disk and analyzed by absorbance spectroscopy (Milton Roy, Spectronic® Genesys^TM^ 5, Rochester, NY, USA) at a wavelength of 610 nm using quartz cuvettes. The photocatalytic performance of the coated ceramics was studied according to the decolorization of indigo carmine using the following equation:

(1) $$Photocatalytic\;efficiency\;=\eta \;\%=\frac{A_{0}-A_{s}}{A_{0}}\times 100$$

where *A_0_* is the initial absorption of the dye and *A_s_* the absorption of the dye after a certain UV irradiation time.

## 3. Results and Discussion 

### 3.1. Physico-Chemical Properties of Ceramic Substrates

The chemical composition of the raw materials used for the production of the ceramic samples is shown in Table 1. The clay mixture (W) presents a typical composition of Ca-rich clay material suitable for the production of white-body ceramics. Ferroalumina (FA) is rich in iron oxide (44.34%) and titanium oxide (5.12%) which is expected to contribute on the photocatalytic efficiency of the coated samples.

The mineralogical compositions of the fired ceramic surfaces are presented in Table 2. For the reference samples (S0) the identified phases are quartz (SiO_2_), albite (NaAlSi_3_O_8_), diopside (CaMgAl_3_Si_2_O_6_), and calcite (CaCO_3_). Hematite (Fe_2_O_3_) was additionally formed in case of S10, S20, and S50 which increases according to the ferroalumina content in the mixes. 

### 3.2. Crystalline Phases and Morphology of Coated Surfaces

**Figure 3:**
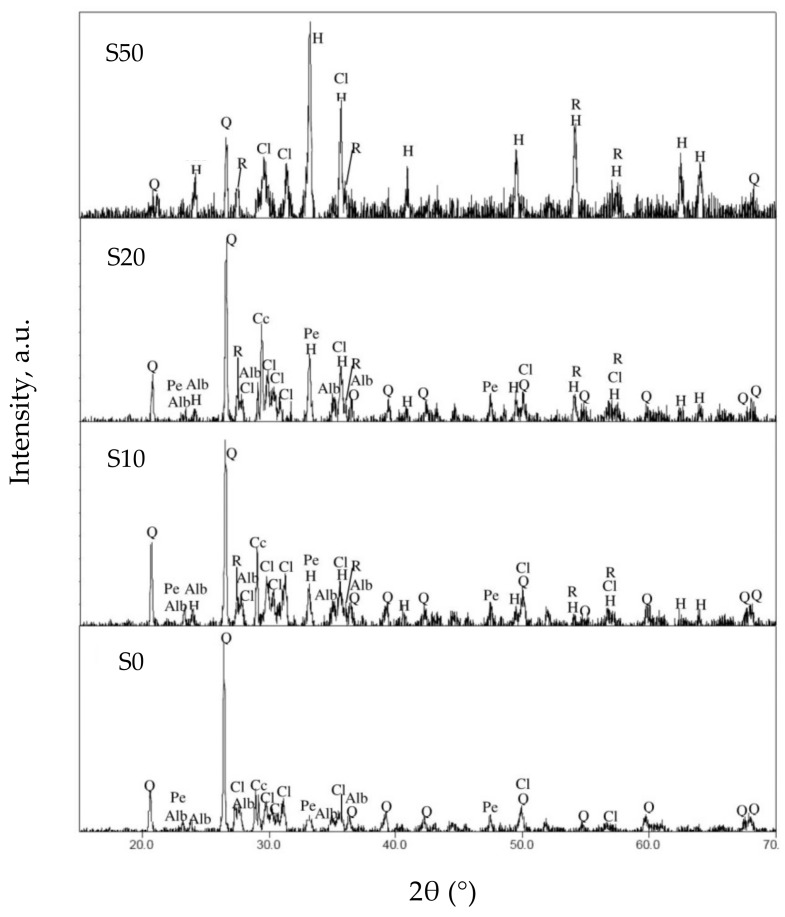
XRD patterns of coated surfaces, after annealing at 600 °C for 1 h. Abbreviations*—* Q: quartz, Alb: albite, Cl: clinopyroxene (aluminium diopside), Cc: calcite, H: hematite, Pe: perovskite, R: rutile.

XRD patterns of the coated and annealed (600 °C) surfaces are shown in Figure 3, whereas the results of the quantitative analysis are presented in Table 3. In all compositions, new formed TiO_2_ phases were identified coexisting with the mineralogical phases of the non-coated ceramics (Table 2). The reference sample (S0) showed the presence of perovskite at 5.2 wt%. For the samples S10 and S20 the formation of mixed-TiO_2_ phases is observed, consisting of rutile (~2.1 wt%) and perovskite (~5.2 wt%). In case of S50, only rutile was detected at 5.7 wt%. The formation of perovskite in the case of S0, S10, and S20 ceramics could be attributed to the presence of albite which act as Ca^2+^ source and reacts with the deposited TiO_2_ during the CVD process. On the other hand, the absence of albite in sample S50 leads only to the deposition of TiO_2_, which after the annealing crystallizes in rutile phase.

**Figure 4:**
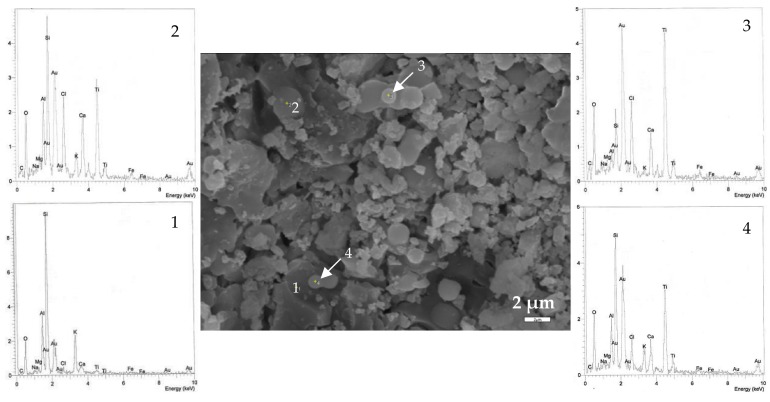
SEM image (4000×) and energy spectrum analysis (**1**–**4**) of the coated reference sample S0, after annealing at 600 °C for 1 h.

**Figure 5:**
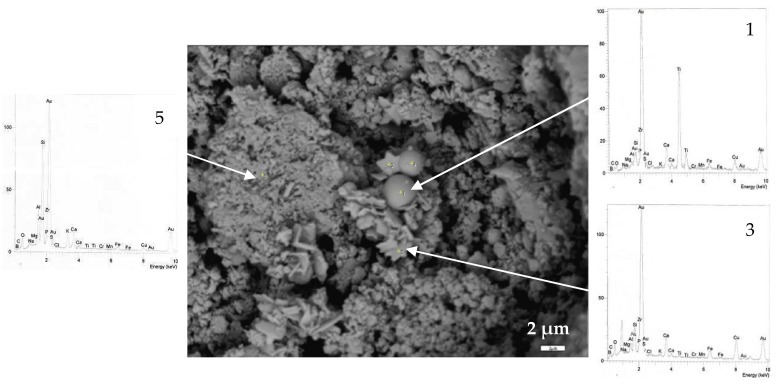
SEM image (3500×) and energy spectrum analysis of coated S10, after annealing at 600 °C for 1 h.

**Figure 6:**
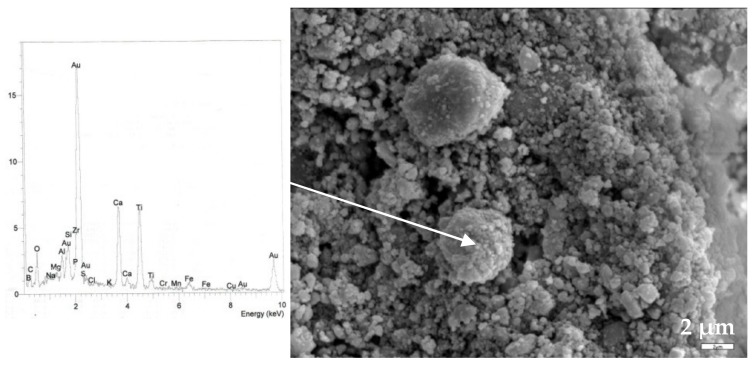
SEM image (4000×) and energy graph of coated surface S20, after annealing at 600 °C for 1 h.

SEM images of coated and annealed (600 °C) ceramic surfaces are presented in Figure 4, Figure 5 and Figure 6. The developed titania coatings on their surfaces is confirmed by EDS analyses revealing that spherical particles are rich in Ti. However, the biggest challenge is to distinguish the different morphologies of TiO_2_ phases, as they were detected in XRD analysis (e.g., rutile and perovskite), both due their small crystal size and the strong interference of the ceramics microstructure (e.g., porosity and microroughness).

The reference sample (S0), Figure 4, exhibits these spherical particles which appear to have been formed sporadically on the ceramics surface with a size up to 2 μm (Figure 4, points 3 and 4). However, it is not unlikely to detect Ti also on other micro-regions, in form of perovskite (CaO∙TiO_2_) as shown from the relative energy graph (Figure 4, (2)); consistent with the XRD results (Table 3). The ratio of Ca and Ti according to the atomic analysis is close to 1:1.

In case of S10, Figure 5, well-formed spherical particles of titania (Figure 5, (1)) have been formed next to flake-like crystals which are rich in Cu, Figure 5, (3), as a result of the doping procedure on the ceramics surfaces. The microstructure presents diversities compared to S0, as small agglomerations are observed among the typical ceramic microstructure closely related to the contained ferroalumina in the raw mix.

The sample S20, Figure 6, shows a different surface morphology compared to the other samples representing a stronger tendency to particle agglomeration attributed to the increased amount of ferroalumina and its related fine particle size. Spherically-formed particles are larger in size, varying from 4 to 8 μm which is typical for low reactor temperatures [15]. EDS analysis confirms the formation of perovskite (Ca:Ti = 1:1). 

### 3.3. TiO_2_ Synthesis

In a low-pressure CVD process the synthesis of TiO_2_ can be represented by the following reaction:

(2) $${\mathrm{TiCl}}_{4}+2{\;H}_{2}O\to {\mathrm{TiO}}_{2}+4\;\mathrm{HCl}$$

The presence of water is important for the hydrolysis of TiCl_4_ and the formation of the TiO_2_ coating. Thus, the surface of the ceramic samples must have the appropriate humidity in order to achieve the desired coating. Moreover, HCl dissolves calcium compounds to CaCl_2_ which is anticipated to react with TiO_2_ in order to form perovskite (CaTiO_3_) that is in agreement with the obtained XRD results (Figure 3).

### 3.4. Photocatalytic Degradation of Indigo Carmine

#### 3.4.1. Preliminary Tests 

**Figure 7:**
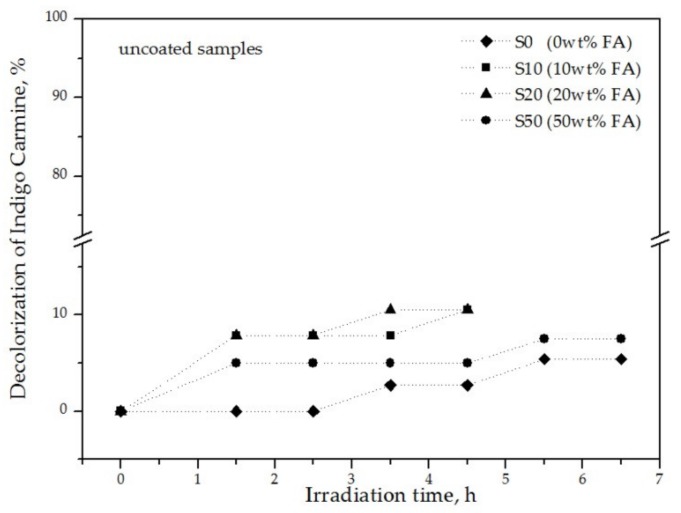
Decolorization ability of uncoated samples as a function of irradiation time.

Preliminary experiments were carried out on the decolorization of indigo carmine solution in order to exclude any possible photolysis due to other parameters apart from the TiO_2_ catalyst. Tests were conducted using a solution of 1 ppm indigo carmine (a) in the absence of any ceramic sample, but under UV irradiation and (b) in the presence of uncoated samples and UV irradiation. Case (a) was performed to see if the dye solution undergoes self-decomposition with UV light and case (b) to ensure that the porosity of the ceramics does not affect the concentration of the dye due to adsorption. The results showed no decolorization of the dye in case of (a). This means that the direct photolysis of IC solution under UV irradiation is excluded, in agreement with [27]. However, in case (b) the samples showed a small decolorization rate, Figure 7, varying from 5.3–10.5%, with the highest value corresponding to the samples S10 and S20; and the lowest to S0. Taking into account the technological properties of the ceramic samples, the water absorption capacity after 24h was found approximately 11%, while the apparent porosity was calculated [26] at 34.7% for S0 and S10, and 39.1% and 41.3% for S20 and S50, respectively. From the obtained results, it can be concluded that the highly porous nature of the samples does not directly influence the decoloring of the dye solution. 

#### 3.4.2. Photocatalytic Activity of Coated Ceramics

**Figure 8:**
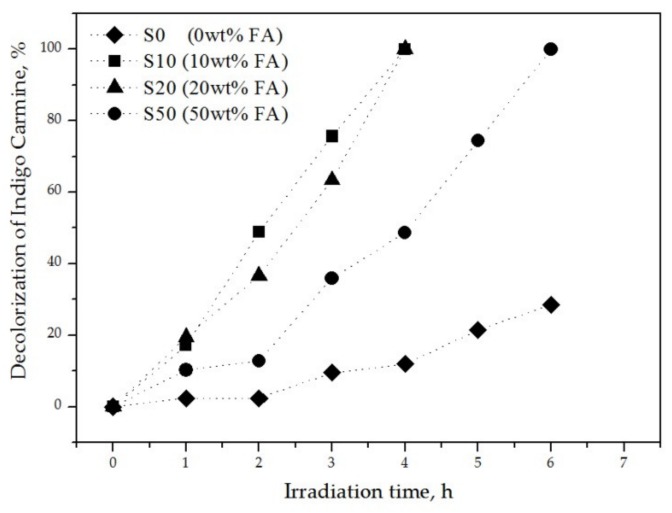
Decolorization (%) of IC solution as a function of irradiation time (h) for all coated samples.

The photocatalytic efficiency of the TiO_2_ coated ceramic samples was examined under UV irradiation and the results are depicted in Figure 8. All coated samples were able to decolorize the dye but at different times and rates. In particular, the best photocatalytic activity was obtained for the samples S10 and S20 as they decomposed the dye 100% after four hours under UV exposure. S50 presented also 100% decomposition of IC solution but at six hours of irradiation. On the contrary, the reference sample S0 showed a significant lower degradation ability of about 29% after six hours of exposure to UV. Comparing the results obtained, it can be concluded that the presence of rutile combined with perovskite (S10 and S20) has the highest photocatalytic activity. Rutile’s activity is adversely affected by the absence of perovskite (S50) only in terms of rate.

## 4. Conclusions

The development of photocatalytic coatings on ceramic substrates with the method of CVD and hydrolysis of TiCl_4_ was successful since promising results were obtained considering the surface characteristics (i.e., the formation of spherical titania spots) and the photodegradation of an organic pollutant. All coated samples presented photocatalytic activity, but at different rates and times. The addition of the industrial by-product ferroalumina as raw material in the clay-body mix seems to favour the degradation ability of the ceramics as the decomposition rate of Indigo Carmine solution reaches 100%. In particular, the most effective ones are the S10 and S20 which decompose at four hours, whereas S50 decomposes at six hours. At the same time, like the latter one, the reference sample was able to decompose only 28% of the dye solution. The correlations between the ferroalumina content in the ceramic samples and the photocatalytic performance is clearly related to the formation of rutile and/or perovskite, the combination of which exhibits the best photocatalytic activity (S10 and S20).

## Figures and Tables

**Table 1:** Chemical analysis of raw materials (in wt%). W: clay mixture and FA: ferroalumina.

	SiO_2_	Al_2_O_3_	CaO	Fe_2_O_3_	MgO	K_2_O	Na_2_O	TiO_2_	L.O.I.
W	48.29	13.61	12.72	5.30	3.11	2.49	0.59	n.d.	13.64
FA	7.79	17.04	11.64	44.34	0.57	0.07	3.17	5.12	9.77

n.d.: not determined.

**Table 2:** Quantitative (wt%) mineralogical composition of the uncoated ceramic samples.

	Quartz	Albite	Calcite	Diopside	Hematite
**S0**	27.9	17.9	14.1	40.1	n.d.
**S10**	22.7	14.4	11.3	45.4	6.2
**S20**	21.3	7.9	10.9	46.4	13.5
**S50**	9.6	n.d.	25.5	20.6	44.2

n.d.: not determined.

**Table 3:** Quantitative (wt%) mineralogical composition of coated ceramics.

	Quartz	Albite	Calcite	Diopside	Hematite	Rutile	Perovskite
**S0**	22.4	18.7	15.1	38.5	n.d.	n.d.	5.2
**S10**	19.3	15.1	14.1	42.1	2.2	2.2	5.1
**S20**	19.0	8.7	13.1	43.6	8.2	2.1	5.3
**S50**	9.0	n.d.	26.6	19.8	38.9	5.7	n.d.

n.d.: not determined
